# Reproducible Large-Scale Isolation of Exosomes from Adipose Tissue-Derived Mesenchymal Stem/Stromal Cells and Their Application in Acute Kidney Injury

**DOI:** 10.3390/ijms21134774

**Published:** 2020-07-05

**Authors:** Jun Ho Lee, Dae Hyun Ha, Hyeon-kyu Go, Jinkwon Youn, Hyun-keun Kim, Richard C. Jin, Randy B. Miller, Do-hyung Kim, Byong Seung Cho, Yong Weon Yi

**Affiliations:** 1 ExoCoBio Exosome Institue (EEI), ExoCoBio Inc., Seoul 08594, Korea; junho.lee@exocobio.com (J.H.L.); dh.ha@exocobio.com (D.H.H.); jinkwon.youn@exocobio.com (J.Y.); hyunkeun.kim@exocobio.com (H.-k.K.); 2 Knotus Co. Ltd., Incheon 22014, Korea; govet@knotus.co.kr; 3 RJ Clinical Institute, Mission Viejo, CA 92691, USA; richard@rjclinical.com; 4 Miller Plastic Surgery, Miami, FL 33133, USA; drmiller@millerplasticsurgery.com

**Keywords:** acute kidney injury (AKI), adipose tissue-derived mesenchymal stem/stromal cells (ASCs), ASC-exosomes, cell-free therapy, large-scale isolation, renal failure, tangential flow filtration (TFF)

## Abstract

Acute kidney injury (AKI) is a fatal medical episode caused by sudden kidney damage or failure, leading to the death of patients within a few hours or days. Previous studies demonstrated that exosomes derived from various mesenchymal stem/stromal cells (MSC-exosomes) have positive effects on renal injuries in multiple experimental animal models of kidney diseases including AKI. However, the mass production of exosomes is a challenge not only in preclinical studies with large animals but also for successful clinical applications. In this respect, tangential flow filtration (TFF) is suitable for good manufacturing practice (GMP)-compliant large-scale production of high-quality exosomes. Until now, no studies have been reported on the use of TFF, but rather ultracentrifugation has been almost exclusively used, to isolate exosomes for AKI therapeutic application in preclinical studies. Here, we demonstrated the reproducible large-scale production of exosomes derived from adipose tissue-derived MSC (ASC-exosomes) using TFF and the lifesaving effect of the ASC-exosomes in a lethal model of cisplatin-induced rat AKI. Our results suggest the possibility of large-scale stable production of ASC-exosomes without loss of function and their successful application in life-threatening diseases.

## 1. Introduction

Acute kidney injury (AKI) or acute renal failure (ARF) is a sudden clinical syndrome of kidney failure or damage that happens within a few hours or days [1]. AKI is manifested by an increase in serum creatinine and a reduction in urine output [1,2], and it is caused by multiple factors, including toxic, ischemic, and immunologic insults, either individually or combined [3,4]. The incidence and mortality of AKI are highly variable from 0.9% to 20% and from 25% to 80%, respectively, depending on the cohorts [4,5]. Since current management strategies for AKI are limited to conservative treatments and waiting for recovery [6], a substantial number of unmet medical needs persist for AKI treatment.

Mesenchymal stem/stromal cells (MSCs) have been applied to reduce the damage caused by renal injuries in preclinical studies [7]. In addition, mounting evidence has demonstrated that exosomes derived from various MSCs (MSC-exosomes) isolated from different tissue sources, such as bone marrow, adipose tissue, umbilical cord tissue, and umbilical cord blood, have therapeutic potential against kidney diseases including AKI and chronic kidney disease (CKD), in multiple animal models [7,8,9]. Exosomes are nano-sized, lipid-bilayered extracellular vesicles (EVs) that are shed by the fusion of multivesicular bodies (MVBs) with the plasma membrane, and they mediate mainly the paracrine effects of stem-cell therapy [10,11]. Interestingly, most of the animal studies into the therapeutic effects of MSC-exosomes in kidney injuries have been performed with exosomes isolated almost exclusively by ultracentrifugation [8,9]. The limitations of ultracentrifugation for exosome isolation include the production of exosomes with co-precipitated contaminants such as protein aggregates, the loss of exosome function because of their aggregation or distortion during the isolation process, and functional inhibition of exosomes by the media used in density gradient ultracentrifugation [12]. Large-scale isolation of single-batch exosomes by ultracentrifugation is also restricted because of the limited instrumental capacity. This raises another problem as new batch of exosomes have to be produced for every experiment. As strict batch-to-batch consistency must be guaranteed for all biological products, ultracentrifugation-based isolation method may not be the optimal method to isolate exosomes for the development of therapeutics.

Many technical challenges need to be overcome in the development of exosome-based therapeutics [13,14]. The large-scale production of high-quality exosomes is the most important factor in their therapeutic application. Among the various isolation methods, tangential flow filtration (TFF) has been proposed as the ideal method for industrial-scale manufacturing of exosomes [12,13,14]. The TFF systems available for good manufacturing practice (GMP) are already in use and provide validated processes and GMP documents [15]. In fact, TFF was first introduced in 2010 for the isolation of exosomes according to size [16] and was gradually employed for exosome isolation or concentration in various experimental settings [17,18,19,20,21,22,23,24,25,26,27,28,29,30,31,32,33,34,35]. More importantly, recent studies demonstrated the superior yield and activity of exosomes isolated by TFF compared with those isolated by ultracentrifugation [26,27,28]. The high-purity isolation of exosomes is achievable with further diafiltration using TFF with appropriate pore sizes and parameters, including transmembrane pressure, flow rate, and the diafiltration factor [36,37]. However, sophisticated optimizations of TFF might be required to preserve the surface-associated proteins which are important functional components of exosomes [38,39]. Interestingly, no studies have used exosomes isolated using TFF for AKI application in animal models so far. Here, we describe the reproducible large-scale production and characterization of exosomes derived from human adipose tissue-derived MSCs (ASC-exosomes) using TFF and the life-saving efficacy of ASC-exosomes in a lethal model of AKI induced by cisplatin in the rat.

## 2. Results

### 2.1. Characterization of ASCs

**Figure 1:**
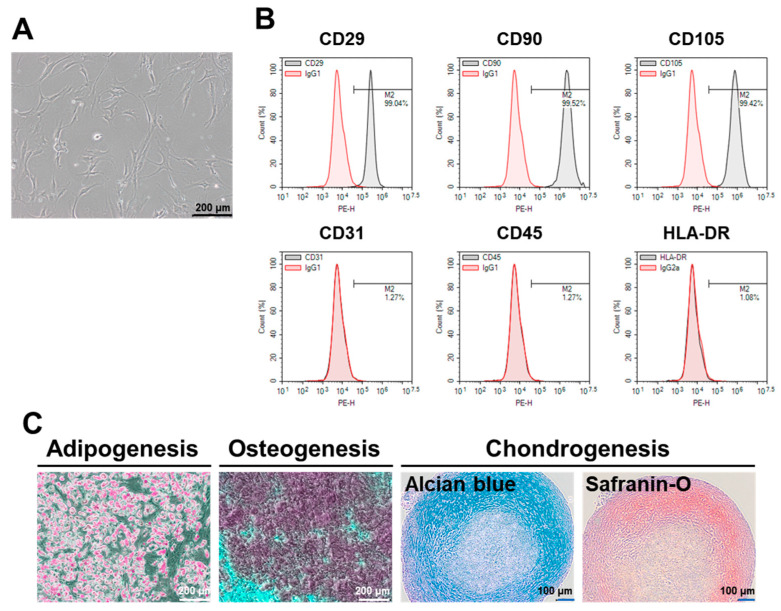
Characteristics of human adipose tissue-derived MSCs (ASCs). (**A**) Representative microscopic image of cultured ASCs demonstrating typical spindle- or fibroblast-like morphology. (**B**) Representative flow cytometry (FCM) data showing the expression of mesenchymal stem/stromal cells (MSC) positive markers (Cluster of Differentiation 29 (CD29), CD90, and CD105) and the absence of negative markers (CD31, CD45, and human leukocyte antigen – DR isotype (HLA-DR)). (**C**) Representative results showing trilineage differentiation of ASCs. Adipocytes, osteocytes, and chondrocytes were detected by Oil Red O, Alizarin red, and Alcian blue and Safranin O staining, respectively.

The characteristics of ASCs recovered from the frozen stock were confirmed by analyzing the morphology, cell surface markers, and trilineage differentiation potentials. The cultured ASCs demonstrated typical spindle- or fibroblast-like shapes and attached to the surface of the plastic culture vessels (Figure 1A). Flow cytometry (FCM) analysis of surface markers confirmed the presence of Cluster of Differentiation 29 (CD29), CD90, and CD105, and the absence of the endothelial marker CD31 and the hematopoietic lineage markers CD45 and human leukocyte antigen – DR isotype (HLA-DR) (Figure 1B). As expected, the ASCs were successfully differentiated into adipocytes, osteocytes, or chondrocytes, each with distinct phenotypes (Figure 1C).

**Figure 2:**
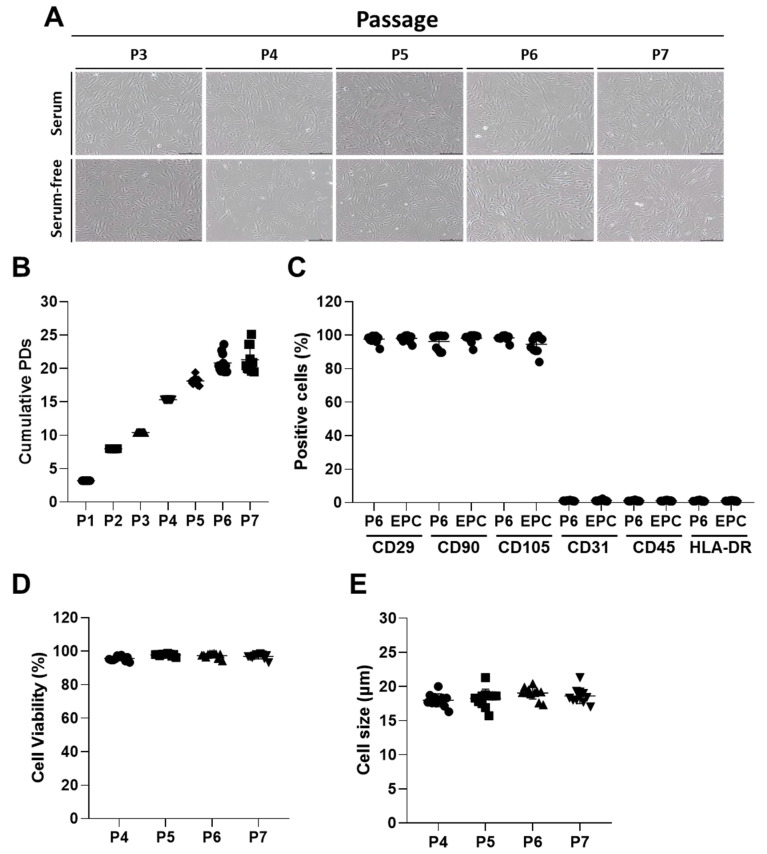
Characteristics of human ASCs over time. (**A**) The morphology of cultured ASCs over time. Scale bar = 200 μm. (**B**) Cumulative population doublings (PDs) of ASCs from the culture of different repeats (*n* = 11). (**C**) The levels of surface markers over time (*n* = 11). (**D**) Viability and (**E**) size of ASCs over time (*n* = 11).

The characteristics of ASCs were further analyzed over time by subculturing up to passage 7. No significant morphological changes in the cultured ASCs were observed up to passage 7 with or without fetal bovine serum (FBS) (Figure 2A). Comparable population doublings were also observed from the cultures of multiple repeats up to passage 7 (Figure 2B). Additionally, cultured ASCs stably expressed all the MSC surface markers (more than 90% of the population) at passage 6 and end of production cells (EPC) after incubation of ASCs at passage 7 with serum-free media to collect the conditioned media of ASCs (ASC-CM) (Figure 2C). In contrast, all the negative markers for ASCs were constantly low (less than 3% of the population) up to passage 6 and EPC (Figure 2C). The viability and size of ASCs were kept highly stable over time (Figure 2D,E). All these results support the stable expansion of the ASCs, which were recovered from frozen stocks, up to passage 7 and EPC after maintenance under serum-free conditions to collect ASC-CM without loss of specific characteristics.

### 2.2. Characterization of ASC-Exosomes

**Figure 3:**
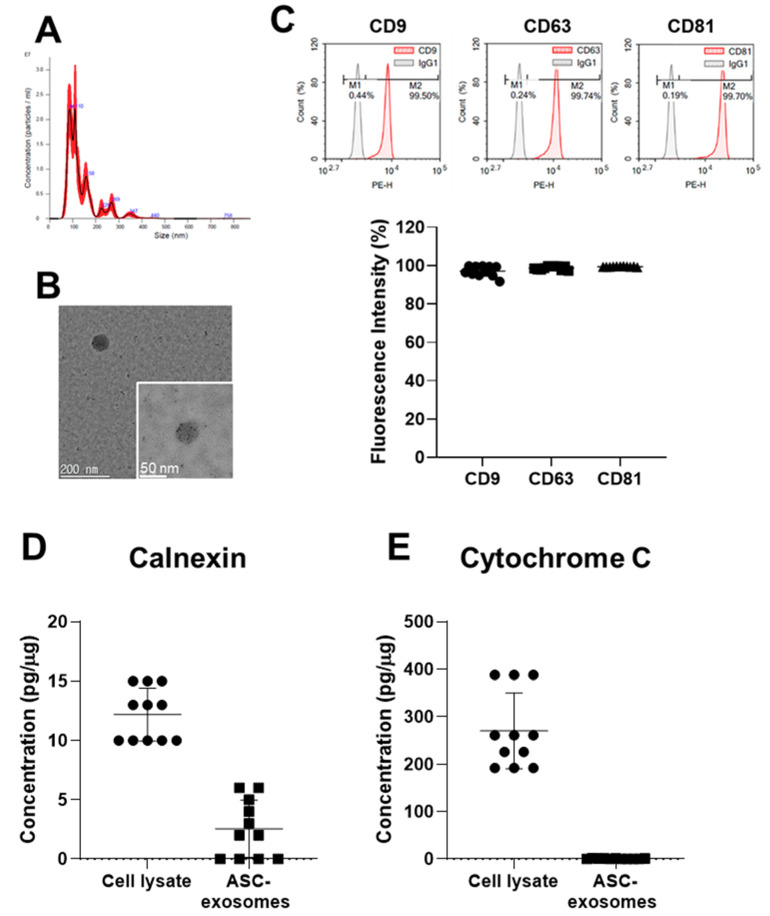
Characteristics of ASC-exosomes produced by the tangential flow filtration (TFF)-based ExoSCRT™ technology. (**A**) Representative nanoparticle tracking analysis (NTA) histogram of particle concentration and size distribution of ASC-exosomes. (**B**) Representative TEM images of ASC-exosomes. (**C**) Representative histograms and cumulative results of FCM analysis of ASC-exosomes (*n* = 11). Amounts of calnexin (**D**) and cytochrome C (**E**) in ASC-exosomes measured by ELISA (*n* = 11).

ASC-exosomes were isolated from more than four liters of ASC-CM by a TFF-based ExoSCRT™ technology [23,34,35] within 4 h. The size distribution and concentration of the isolated ASC-exosomes were analyzed by nanoparticle tracking analysis (NTA). The size of the ASC-exosomes was ranged from 30 to 200 nm with a mean value of 168 ± 33 nm and mode value of 108 ± 20 nm (Figure 3A). Transmitted electron microscopy (TEM) analysis revealed the spherical morphology of the ASC-exosomes (Figure 3B). FCM analysis showed the ASC-exosomes expressed exosomal markers CD9, CD63 and CD81, at comparable levels across multiple batches. However, the levels of calnexin and cytochrome C were negligible in the multiple different batches (Figure 3D,E). These results suggest that the TFF-based ExoSCRT™ technology facilitated the reproducible production of ASC-exosomes of a stable size and identity, and with negligible cellular impurities.

### 2.3. Reproducible Production of ASC-Exosomes by TFF

**Figure 4:**
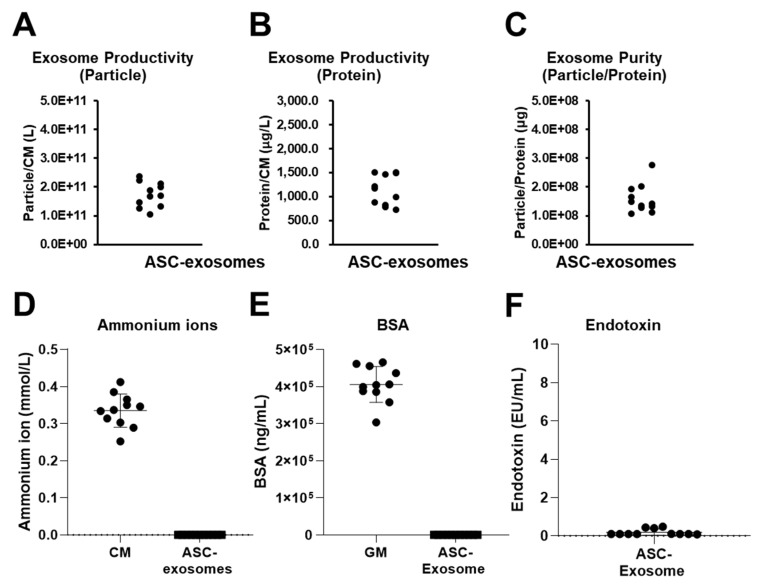
Reproducibility of TFF isolation of ASC-exosomes. Productivities of ASC-exosomes as (**A**) particles from 1 Liter of ASC conditioned media (ASC-CM) and (**B**) microgram of proteins from 1 Liter of ASC-CM (*n* = 11). (**C**) Purities of ASC-exosomes (*n* = 11). Levels of ammonium ions (**D**), bovine serum albumin (BSA) (**E**), and endotoxin (**F**) in multiple ASC-exosomes preparations (*n* = 11).

Although TFF was introduced 10 years ago to isolate exosomes from large volumes of fluids, limited studies have been performed on the reproducibility of the method and functionality of the resulting exosomes [20,26,28,29,31]. We further analyzed multiple aspects of the reproducibility of the ExoSCRT™ technology. Exosome productivity can be defined as the number of isolated exosomes from a unit volume of conditioned media (CM). Productivity can also be defined as the concentration of an isolated exosomal protein from a unit volume of CM. As shown in Figure 4A,B, the productivities of ASC-exosomes from different batches ranged from 1.05 × 10^11^ to 2.36 × 10^11^ particles per liter of CM and from 720 to 1507 microgram per liter of CM. These values are comparable to those of a recent publication [26]. The purities (particles per microgram) in multiple batches of isolated ASC-exosomes ranged from 1.07 × 10^8^ to 2.77 × 10^8^ (Figure 4C). 

Ammonium ion is well-known cellular wastes products. Sub-millimolar levels of ammonium ions were detectable in the various preparations of the ASC-CM (Figure 4D). The levels of this waste product markedly dropped in the isolated ASC-exosomes, leading to almost undetectable levels in the multiple batches of ASC-exosomes (Figure 4D). The residual amount of bovine serum albumin (BSA) was also determined. About 400 pg/mL of BSA was detected in ASC growth media containing FBS (Figure 4E). However, BSA levels were remarkably reduced to below 50 pg/10^8^ particles of ASC-exosomes. According to World Health Organization (WHO) guidance, the concentration of BSA should be no greater than 50 ng per dose of vaccine for humans because of the potential of allergic reactions [40]. In addition, the endotoxin levels were below 0.19 EU/10^8^ particles of ASC-exosomes in the multiple batches (Figure 4F).

**Figure 5:**
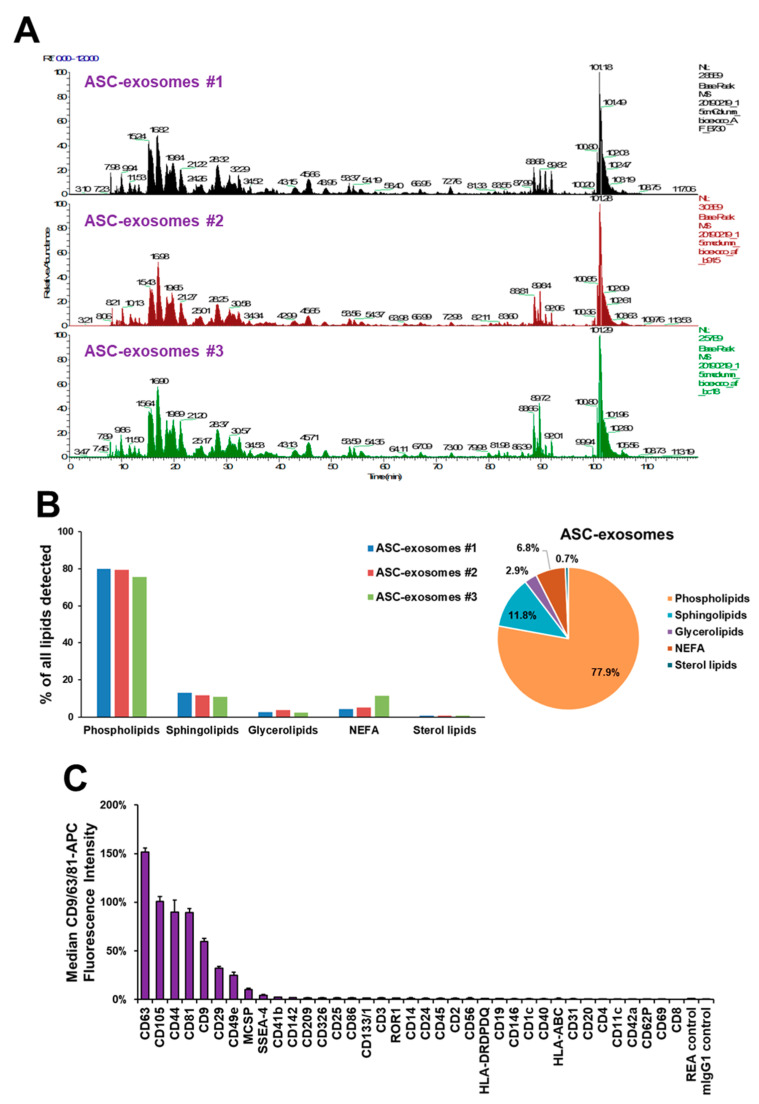
Profiling of ASC-exosomes. (**A**) Proteomic profiling of ASC-exosomes (*n* = 3). (**B**) Profiling of lipids in ASC-exosomes (*n* = 3). (**C**) Multiplex profiling of ASC-exosomes surface proteins. The graph shows the normalized CD9/CD63/CD81-APC signaling intensities (%) from the beads conjugated with 39 antibodies against indicated surface proteins after background correction. Data are presented as mean ± SEM (*n* = 11).

The characteristics of the ASC-exosomes were further analyzed by profiling for proteomes, lipids, and surface proteins. As shown in Figure 5A, the base peak profiles of LC-MS/MS proteomic analysis for three batches of ASC-exosomes were quite comparable. A database search resulted in 471 proteins (46.7%) that were common out of a total 1008 proteins identified from the three batches of ASC-exosomes [34]. However, further studies are needed to evaluate the reproducibility of this analysis, since proteomic analysis is limited by inherent variabilities [41,42].

A total of 373 lipid species were identified in ASC-exosomes [34], and their relative ratios were quite comparable (Figure 5B). Phospholipid species were the most abundant lipids in the ASC-exosomes, followed by sphingolipids, glycerolipids, non-esterified fatty acids (NEFA), and sterol lipids. Although the lipid composition of ASC-exosomes was similar to that of ASCs, the ratios of sphingolipids and NEFA to total lipids were higher in ASC-exosomes than ASCs [34]. 

The ASC-exosomes were further analyzed for surface markers, as the profile of surface markers may represent a degree of exosome variability [43]. Analysis of 11 batches of ASC-exosomes resulted in a high degree of consistency in the surface marker protein profile (Figure 5C). As expected, the well-known exosomal markers CD63, CD9, and CD81 were relatively abundant. An MSC marker, CD105, as well as CD44, CD29, CD49e, and melanoma-associated chondroitin sulfate proteoglycan (MCSP) were found on the surface of ASC-exosomes. Previously, CD44 was reported to be important for directing MSC within the damaged kidney [44]. In addition, MSC-exosomes were found to be preferentially distributed in the damaged kidneys of mice with glycerol-induced AKI compared to in the health kidneys of control mice [45]. Taken together, it is highly plausible that ASC-exosomes are targeted towards damaged kidneys in a CD44-dependent manner in animals with AKI. 

### 2.4. Protection of Animals from Death due to Cisplatin-Induced AKI by ASC-Exosomes

**Figure 6:**
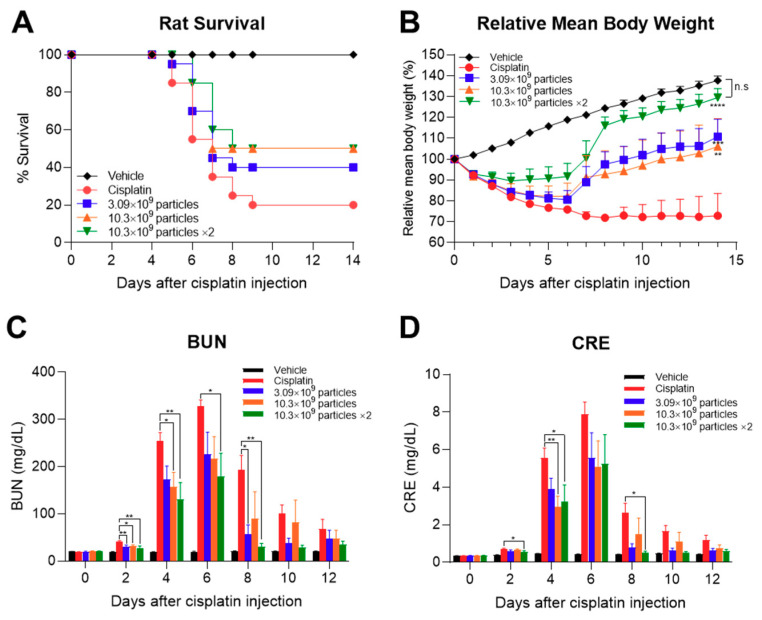
Therapeutic effects of ASC-exosomes on cisplatin-induced AKI in rats. (**A**) Number of surviving animals over time. (**B**) Changes in relative body weight over time. The levels of (**C**) blood urea nitrogen (BUN) and (**D**) serum creatinine over time. Results are presented as mean ± SEM. *n* = 10 for each group. *p < 0.05, **p < 0.01 and ***p < 0.001 vs cisplatin control group. Vehicle: normal rat injected with vehicle control; cisplatin: cisplatin-challenged rat injected with vehicle control; other: cisplatin-challenged rat injected with indicated dose per head of ASC-exosomes; x2: two times administration of ASC-exosomes on day 0 and 2.

We next evaluated the function of TFF-isolated ASC-exosomes in an AKI animal model. Since AKI is a life-threatening syndrome, we selected a lethal AKI model to confirm the function of ASC-exosomes in extremely harsh conditions. Cisplatin induces AKI in both humans and experimental animals, including rats and mice [46,47]. AKI was induced by intraperitoneal (IP) administration of 10 mg/kg cisplatin in Sprague-Dawley (SD) rats. ASC-exosomes were administered by intravenous (IV) injection through the tail vein at 8 h after cisplatin injection. For repeated administration, ASC-exosomes were further injected 48 h after the first dose. As shown in Figure 6A, AKI-induced death of the animals began 5 days after cisplatin challenge and resulted in an 80% death rate in the vehicle- -treated group. In contrast, an increase ASC-exosomes saved the rats from cisplatin-induced death, with up to 50% survival (Figure 6A). More interestingly, the ASC-exosomes retarded the loss of body weight due to cisplatin-induced AKI (Figure 6B). Repeated administrations of high-dose ASC-exosomes eventually restored the body weights of animals to near those of the uninduced normal controls. As expected, blood urea nitrogen (BUN) and serum creatinine levels were also reduced by ASC-exosomes in a dose-dependent manner, resulting in near-normal levels in the surviving animals (Figure 6C,D). A limitation of the current study is the absence of histological and molecular biological analyzes, which could not be performed because of the lethal model study design. Although further histological analyzes should be performed in a separate study that does not employ the lethal model, these results suggest that TFF-manufactured ASC-exosomes saved animals from lethal injury of the kidneys. In fact, this is the first report showing that ASC-exosomes have a life-saving effect in AKI animal models. 

## 3. Discussion

Until now, there has been no approved therapeutic intervention available for AKI [48]. Various toxic or ischemic insults lead to tubular injury in AKI through multiple pathways, including microvascular dysfunction, oxidative stress, inflammation, immune dysregulation, cell death, and/or senescence [49]. Numerous potential monotherapies targeting individual pathways are under clinical development [48]; however, the targeting of multiple pathways is preferable to alleviate or inhibit the progression of complex diseases [50].

Recently, kidney cells have been revealed to be capable of regenerating and repairing themselves throughout their lifetimes, which is in contrast to the traditional notion of the kidney as a static organ with limited cellular turnover and regenerative capacity [4,51]. In fact, recovery from AKI depends on the regenerative capacity of renal tubules [4]; the failure to replace injured tubular epithelial cells during recovery may lead to fibrosis and CKD [52]. In this context, new therapeutics aimed at providing regenerative potential in addition to targeting multiple pathways might be the most promising form of AKI treatment.

It is now widely accepted that MSC-exosomes are the next-generation of regenerative therapeutics capable of targeting multiple pathways with regenerative function and overcoming the limitations of cell-based therapeutics [14,53,54]. Several studies have already demonstrated the therapeutic effects of MSC-exosomes from different cell sources, including bone marrow, Wharton’s Jelly, and umbilical cords, in a diversity of AKI animal models [7,8,9]. For instance, injection of human umbilical cord-MSC (UC-MSC)-derived exosomes reduced apoptosis and necrosis of proximal kidney tubules by ameliorating oxidative stress in a cisplatin-induced rat AKI model. In vitro, UC-MSC-exosomes induced proliferation of a renal tubular epithelial cell line and suppressed expression of caspase 3 and the authors show that these effects were mediated by activation of extracellular-signal-regulated kinase (ERK) 1/2 pathway [55]. Another study by Zhang et al. shows that human Wharton’s jelly-MSC-derived extracellular vesicles protected kidney function of rats in ischemia-reperfusion injury (IRI) model by reducing oxidative stress via activation of nuclear factor erythroid 2-related factor 2 (NRF2) [56]. In fact, NRF2 was reported as a cargo protein of ASC-exosomes which reduced the high glucose-induced premature senescence of endothelial progenitor cells [57]. In addition, antioxidant enzymes peroxiredoxin (PRDX) 1, 4, and 6 have been found in the proteome of ASC-exosomes by our group [34]. These results suggest that the anti-oxidative activity of ASC-exosomes might contribute the protection of life from lethal renal injury. Further study will be needed to decipher the molecular mechanism of this with GMP-grade ASC-exosomes for clinical translation. As observed in other disease models, various miRNA cargoes, such as miR-30, miR-199a-5p, miR-486-5p in MSC-exosomes also contribute to the protective and renal-regenerative effects in AKI models as well [58,59,60]. However, the clinical translation of these studies is limited by available exosome-isolation methods; ultracentrifugation-based isolation methods were applied in all of the 31 reported studies on preclinical rodent models [9]. Ultracentrifugation is the most widely used method for isolating exosomes from the MSC-CM [61]. However, TFF-based methods are recognized as the most suitable methods for the manufacture of GMP-grade exosomes from large volumes of CM, with comparable high yield and purity as size exclusion chromatography-based methods [12,13,14,62]. 

In the present study, we demonstrated the stable production of ASC-exosomes from cryopreserved ASCs. ASCs could be expanded up to passage 7 without loss of specific characteristics. The characteristics of ASCs were also maintained after incubation with serum-free media to obtain the CM. Using this process, several liters of CM could be obtained and further processed to isolate ASC-exosomes with a TFF-based method. Analysis of multiple batches of isolated ASC-exosomes demonstrated (1) the presence of stable characteristics, including size and surface markers; (2) the efficient removal of the cellular waste product (ammonium ion) and process impurity (BSA); and (3) the reproducibility and purity of the ASC-exosomes. Proteomic and lipidomic profiling analyzes resulted in comparable profiles in three batches of ASC-exosomes. More importantly, multiplex surface-marker profiling analysis successfully demonstrated a high degree of consistency in the levels of 37 surface-marker proteins in 11 different batches of ASC-exosomes. Overall, the established TFF isolation method allows for the reproducible production of exosomes with stable characteristics from large volume of culture media.

Previously, ASC-exosomes, isolated via ultracentrifugation, were reported to protect rat kidneys from acute ischemia-reperfusion injury [6]. However, the therapeutic effects of exosomes might vary between isolation methods because the integrity of the isolated exosomes and the molecular composition of their cargo and/or surface-associated molecules differ among the isolation methods. Thus, ASC-exosomes isolated via TFF may show different cargo profiles from ASC-exosomes isolated via ultracentrifugation and different mode of actions in alleviating AKI. 

**Figure 7:**
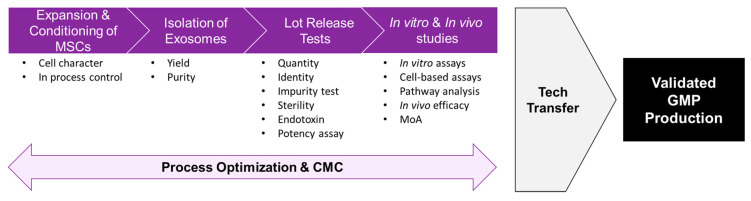
Schematic diagram of process development for exosome-based therapeutics.

The present study successfully evaluated, for the first time, the life-saving efficacy of ASC-exosomes isolated by TFF in a lethal model of AKI induced by cisplatin-challenge in rats. As this study mainly focused on the manufacturing aspect of ASC-exosomes, ASC-exosomes were applied in an extreme, lethal model of AKI to observe their protective and life-saving efficacies. To further delineate the underlying molecular mechanisms, the effects of ASC-exosomes must be tested in AKI model induced by sub-lethal dose of cisplatin and additional factors, such as renal histology, infiltration of immune cells and signaling pathways relevant to cell death, must be analyzed. Although further studies are needed to decipher the underlining molecular mechanisms, the isolation of ASC-exosomes by TFF might provide a scalable GMP process that facilitates the development of exosome-based therapeutics in the near future (Figure 7). 

## 4. Materials and Methods

### 4.1. Kits and Reagents

The reagents used in this study were purchased from the following sources: high glucose Dulbecco’s modified Eagle’s medium (DMEM), fetal bovine serum (FBS), penicillin-streptomycin, Dulbecco’s phosphate buffered saline (DPBS), Trypsin-EDTA, L-glutamine, sodium pyruvate, and Exosome-Human CD81 Flow Detection Reagent from Thermo Fisher Scientific (Carlsbad, CA, USA); differentiation medium for adipogenic, osteogenic, and chondrogenic lineage from Cefo Co., Ltd.(Seoul, Korea) or Thermo Fisher Scientific (Carlsbad, CA, USA); normal IgG, Phycoerythrin (PE)-conjugated antibodies for CD29, CD90, CD105, CD31, CD45, HLA-DR, CD9, CD63, and CD81 from BD Biosciences (San Jose, CA, USA); Pyrogent™ Gel Clot Limulus Amebocyte Lysate (LAL) assay for the bacterial endotoxin test from Lonza (Morristown, NJ, USA); e-Myco™ Mycoplasma PCR Detection Kit from iNtRON Biotechnology (Seongnam-si, Gyeonggi-do, Korea); ELISA for bovine serum albumin (BSA) from Shibayagi (Gunma, Japan); ELISA for calnexin from LSBio (Seattle, WA, USA); ELISA for cytochrome C from Abcam (Cambridge, UK) or R&D Systems (Minneapolis, MN, USA); detection reagents for ammonium ions from Roche Diagnostics (Mannheim, Germany); MACSPlex Exosome kit (human) from Miltenyi Biotec (Bergish Gladbach, Germany); 500-kDa molecular weight cut-off filter membrane cartridge from GE Healthcare (Chicago, IL, USA) or Pall (Port Washington, New York, USA); and cisplatin from Sigma Aldrich (St. Louis, MO, USA). 

### 4.2. Culture and Characterization of ASCs

A human ASC cryostock of passage 4 was prepared as described previously [34] and stored in liquid nitrogen. Frozen cells were thawed at 37 °C, plated at a density of 3000 cells/cm^2^, and cultured with DMEM containing 10% FBS and 1% penicillin-streptomycin at 37 °C and 5% CO_2_. Cultured ASCs were sub-cultured before the cultures reached confluence. The viability and size of the cells were monitored by an automated cell counter with trypan blue staining [63]. ASCs were characterized for surface-marker expression and trilineage differentiation potentials according to the criteria described by the International Society of Cellular Therapy [64].

### 4.3. Isolation and Characterization of ASC-Exosomes

To obtain ASC-CM, a vial of ASC stock was thawed and sub-cultured with gradually increasing culture scales in a T175 flask and a 1- or 2-layered Cell Factory Systems (Thermo Fisher Scientific; Carlsbad, CA, USA) before passage 7 at 37 °C and 5% CO_2_. At passage 7, the ASCs were plated at a density of 6000 cells/cm^2^ in 10-layered Cell Factory Systems and cultured up to 90% confluency in DMEM containing 10% FBS at 37 °C and 5% CO_2_. The ASCs were washed three times with DPBS to remove FBS and supplemented with serum- and phenol-red-free DMEM containing 1% L-glutamine (200 mM) and 1% sodium pyruvate (100 mM). The cells were further incubated for 24 h at 37 °C and 5% CO_2_ before the CM were collected. 

ASC-exosomes (ASCE™, ASCE is the proprietary trademark of ExoCoBio) were isolated from ASC-CM using the TFF-based ExoSCRT™ technology as previously described [23,34,35]. Briefly, to remove larger non-exosomal particles, including cells, cell debris, microvesicles, and apoptotic bodies, the ASC-CM were filtered through a 0.22-μm polyethersulfone membrane filter (Merck Millipore, Billerica, MA, USA) and then concentrated by TFF with a 500 kDa molecular weight cut-off filter. The concentrated ASC-CM was further diafiltrated with appropriate volumes of PBS to remove non-exosomal proteins, nutrients, and cellular waste products such as lactate and ammonia. Isolated ASC-exosomes were stored at –80 °C as small aliquots in sterile polypropylene tubes. The frozen ASC-exosomes were stored at 4 °C until completely thawed before further use. 

Characterization of the ASC-exosomes was performed according to the Minimal Information for Studies of Extracellular Vesicels 2018 (MISEV2018) recommended by the International Society for Extracellular Vesicles [65]. NTA was performed with a NanoSight NS300 (Malvern Panalytical, Amesbury, UK) as described previously [23,34,35]. TEM analysis, FCM analysis, and protein quantification were also conducted as described previously [23,34,35]. ELISAs for calnexin, cytochrome C, and BSA were performed according to the manufacturer’s recommendations. Measurements of ammonium ions were performed according to the manufacturer’s instructions. Proteomic and lipidomic analyses of ASC-exosomes were conducted as described previously [34]. 

### 4.4. Animal Study

The animal study was approved by the Knotus Institutional Animal Care and Use Committee (IACUC; approval number 17-KE-315, November 10, 2017) and performed according to the Animal Experimentation Policy of Knotus Co., Ltd. (Incheon, Korea). Six week-old male SD rats were obtained from Orient Bio (Seongnam, Gyonggi-do, Korea) and kept under controlled environmental conditions (temperature: 23 ± 3 °C; relative humidity: 55 ± 15%; ventilation: 10–20 air changes/hr; and luminous intensity: 150–300 Lux) with a 12-h light-dark cycle in the experimental animal facility at Knotus. AKI was induced by IP injection of 10 mg/kg cisplatin that was dissolved in saline as described previously [66]. At 8 h after cisplatin injection, the vehicle control or the increased amounts of ASC-exosomes were IV administered at a flow rate of 1 mL/min through the tail vein. For repeated injection, ASC-exosomes were further administered 48 h after the first injection. The amounts of ASC-exosomes were 3.09 × 10^9^ and 10.3 × 10^9^ particles/head (corresponding to 21 and 70 μg of protein, respectively). The animals’ body weights and survival were monitored daily. Blood samples were collected on day 0 (before cisplatin injection), 2, 4, 6, 8, and 12, and analyzed for blood urea nitrogen (BUN) and creatinine (CRE) with the 7180 Clinical Analyzer (Hitachi High-Technologies, Tokyo, Japan). 

### 4.5. Statistical Analysis

Prism 8.0 (GraphPad Software Inc., San Diego, CA, USA) was used to analyze data. Comparisons among different groups were performed by one-way analysis of variance (ANOVA) followed by Dunnett’s multiple-comparison of multiple means. All values are expressed as mean ± SEM. *p*-values of *p < 0.05, **p < 0.01, and ***p < 0.001 were considered statistically significant.

## Abbreviations

AKI	acute kidney injury
ANOVA	analysis of variance
ARF	acute renal failure
ASCs	adipose tissue-derived MSCs
ASC-CM	ASC conditioned media
ASC-exosomes	exosomes derived from ASCs
BSA	bovine serum albumin
BUN	blood urea nitrogen
CKD	chronic kidney disease
CM	conditioned media
CRE	creatinine
DMEM	Dulbecco’s Modified Eagle’s Medium
DPBS	Dulbecco’s Phosphate Buffered Saline
EDTA	Ethylenediaminetetraacetic acid
ELISA	enzyme-linked immunosorbent assay
EPC	End of production cells
ERK	extracellular-signal-regulated kinase
EVs	extracellular vesicles
FBS	fetal bovine serum
FCM	flow cytometry
GMP	good manufacturing process
HLA	human leukocyte antigen
IP	intraperitoneal
IRI	ischemia-reperfusion injury
IV	intravenous
MISEV	Minimal Information for Studies of Extracellular Vesicels
MSCs	mesenchymal stem/stromal cells
MSC-exosomes	exosomes derived from MSCs
MVBs	multivesicular bodies
NEFA	non-esterified fatty acids
NRF2	nuclear factor erythroid 2-related factor 2
NTA	nanoparticle tracking analysis
PDs	population doublings
PRDX	peroxiredoxin
SD	Sprague-Dawley
TEM	Transmitted electron microscopy
TFF	tangential flow filtration
WHO	World Health Organization
